# NRP1 and synapse formation

**DOI:** 10.18632/oncotarget.13462

**Published:** 2016-11-18

**Authors:** Fabrice Ango

**Affiliations:** Department of Neurobiology, CNRS, UMR5203, Institut de Génomique Fonctionnelle, Montpellier, France; INSERM, U1191, Montpellier, France; Université de Montpellier, Montpellier, France

**Keywords:** axon initial segment, GABA, neurofascin186, semaphorin, adhesion

Perception and behavior are critically dependent on synaptic communication between specific neuronal types. Understanding how neurons achieve such a “synaptic specificity” is therefore one of the most fundamental issues in developmental neuroscience. The final lock between the pre- and post-synaptic elements is the culmination of a multiple-step program that includes cell migration, cell differentiation, axon guidance, cell type recognition, subcellular innervation and synapse stabilization. While each of these steps might certainly happen independently, the mechanism that controls the transition between them remains to be explored.

Local circuit development can be taken as a model for understanding the orchestration of synaptogenesis. Thus the question of whether local GABAergic axons are actively guided by local cues towards their target or selectively pruned is largely debated. In cerebellar cortex, Purkinje cells receive specific inhibitory inputs ending on the soma and axon initial segment (AIS) from GABAergic Basket cells (BCs) to form the so called “pinceau synapse”. In previous studies, we and others showed that during development, once basket axon collaterals reach Purkinje cell soma, the subcellular gradient of NF186, a Cell Adhesion Molecule (CAM) of the L1-CAM family, directs basket axon terminals to Purkinje cell AIS and the ankyrinG-associated form of NF186 at AIS is necessary for pinceau synapse formation and/or stabilization [1, 2]. Indeed, knocking down of the scaffolding protein Ankyrin-G or NF186 disrupts pinceau synapse stabilization, even in adult mouse. However the mechanisms that steer BC axons towards PC somata and coordinate local NF186 binding to BCs remain unknown.

To investigate this issue, we conducted a survey of all classical axon guidance molecules (i.e. Ephrins, Netrins, Slits and Semaphorins families) expressed by PCs during cerebellar development [3]. Our data identified the axon guidance molecule Semaphorin-3A (Sema3A) as the main PC secreted cues. Knockdown of Sema3A or its obligatory binding receptor subunit NRP1 induced aberrant BC axon organization and reduced terminal axon branching [4, 5].

In the present study we further show that Sema3A and NRP1 are the main molecular cues that coordinate AIS innervation by controlling both BC axons pathfinding and subcellular target recognition [4]. In particular, NRP1 expressed in BC axons plays the role of matchmaker or facilitator, first by bringing the pre- and post-synaptic elements in close proximity and second by sealing their physical interaction through a direct binding with the post-synaptic molecule NF186. The first step relies on two forms of Sema3A: a secreted-Sema3A form that controls BC axon attraction and an extracellular matrix-or cell-attached-Sema3A form that stabilizes NRP1 at the axonal surface in close proximity to PC soma and AIS. Then, subcellular domain recognition and innervation is mediated by stabilized NRP1 through trans-synaptic interaction of the molecule with NF186. Interestingly, the binding of Sema3A and NF186 to NRP1 appeared as non-mutually exclusive, suggesting that a ternary complex formed by these molecules might facilitate the initial contact between the pre- and post-synaptic partners. This example of how axons choose their post-synaptic targets is confirmed by both *in vitro* and *in vivo* experiments that clearly revealed the need of both Sema3A and NF186 cooperation via presynaptic NRP1 for axonal innervation [4]. Thus by orchestrating axon guidance, recognition and innervation, pre-synaptic NRP1, provides a smooth transition between each of these steps. This alleviates some of the burden of finding a minute piece membrane such as the AIS among various local possibilities. In the end, our data support one of the most intuitive models for pre- and post-synaptic assembly, by using matched expression of heterophilic cell-surface molecules. However, the final assembly needs an external switch to locally stabilize the recognition cues, suggesting that match CAM expression patterns in central nervous system will not necessary cause pre- and post-synaptic assembly.

The chemo-affinity theory proposed by Sperry states that different cells bear distinct cell-surface proteins that serve as markers [6], and the initial hypothesis required a large number of proteins to code such a specificity. In a developmental perspective, the need for such a complex coding might be moderate due to the fine regulation of the multiple steps preceding the final lock between the pre-and the post-synaptic sites.
